# Supplementary material for: Identification of photoperiod-induced specific miRNAs in the adrenal glands of Sunite sheep (Ovis aries)
Source: Front Vet Sci. 2022 Jul 22;9:888207. doi: 10.3389/fvets.2022.888207 (PMC9354845; doi:10.3389/fvets.2022.888207)
Supplement: Supplementary file 1 [file Data_Sheet_1.ZIP › Supplementary file/miRDeep2 file.zip!/miRDeep2 file/Know_Structure/LP42A1/oar-mir-495.pdf]

miRBase precursor : oar-mir-495
Total read count : 9515
oar-miR-495-5p read count : 59
oar-miR-495-3p read count : 9438
remaining reads : 18

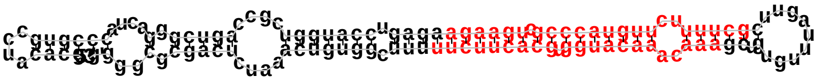

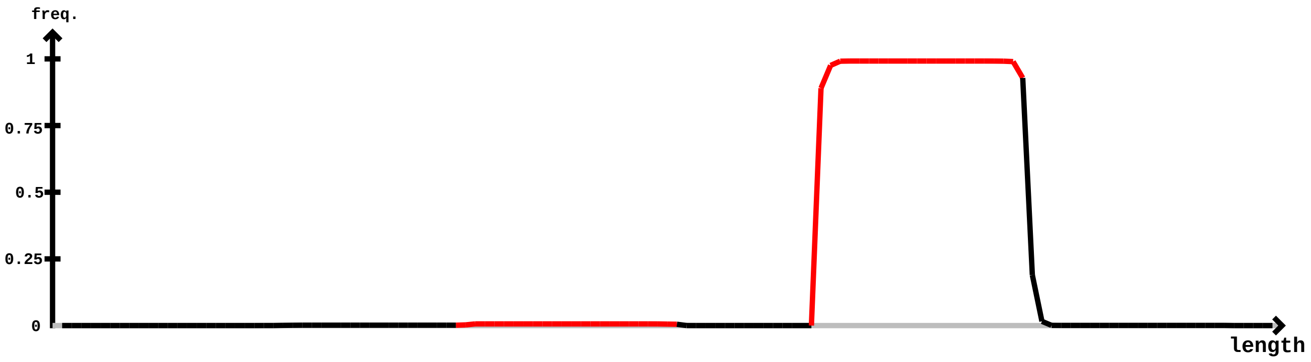

oar-miR-495-3p

oar-miR-495-5p

```
5'- gcacauccgugcccaucagggcugaccgcugguaccugagaagaagucgcccauguucuuuucgcuugauuugugucgaaacaaacauggugcacuucuuuuucggugucaaaucucagcgcgggg  -3'   exp
    ((((....))))((....(.(((((....(((((((.((((((((((.(((((((((..((((((.........).)))))..))))))).)))))))))))).)))))))....))))).)..))   reads   mm
    ....................gcugaccgcugguaccugag..............................................................................   1       0
    ......................ugaccgcugguaccugagaa....................................................................................   6       0
    .......................gaccgcugguaccugagaa....................................................................................   3       0
    ........................accgcugguaccugagaa....................................................................................   3       0
    .........................................agaagucgcccauguucuuu.................................................................   1       0
    .........................................agaagucgcccauguucuuuu................................................................   3       0
    .........................................agaagucgcccauguucuuuuc...............................................................   1       0
    .........................................agaagucgcccauguucuuuucg..............................................................   7       0
    ..........................................gaagucgcccauguucuuuu................................................................   3       0
    ..........................................gaagucgcccauguucuuuuc...............................................................   4       0
    ..........................................gaagucgcccauguucuuuucg..............................................................   39      0
    ..........................................gaagucgcccauguucuuuucgc.............................................................   1       0
    ..............................................................................aaacaaacauggugcacu..............................   1       0
    ..............................................................................aaacaaacauggugcacuA.............................   1       1
    ..............................................................................aaacaaacauggugcacuu.............................   3       0
    ..............................................................................aaacaaacauggugcacuuc............................   15      0
    ..............................................................................aaacaaacauggugcacuucu...........................   543     0
    ..............................................................................aaacaaacauggugcacuucuu..........................   6687    0
    ..............................................................................aaacaaacauggugcacuucuuu.........................   1177    0
    ..............................................................................aaacaaacauggugcacuucuuuu........................   42      0
    ...............................................................................aacaaacauggugcacuucu...........................   39      0
    ...............................................................................aacaaacauggugcacuucuu..........................   331     0
    ...............................................................................aacaaacauggugcacuucuuu.........................   423     0
    ...............................................................................aacaaacauggugcacuucuuuu........................   19      0
    ...............................................................................aacaaacauggugcacuucuuuuu.......................   1       0
    ................................................................................acaaacauggugcacuucu...........................   1       0
    ................................................................................acaaacauggugcacuucuu..........................   15      0
    ................................................................................acaaacauggugcacuucuuu.........................   55      0
    ................................................................................acaaacauggugcacuucuuuu........................   79      0
    .................................................................................caaacauggugcacuucuu..........................   1       0
    .................................................................................caaacauggugcacuucuuuu........................   4       0
    .................................................................................caaacauggugcacuucuuuuu.......................   1       0
    ....................................................................................................uuucggugucaaaucucagcg.....   5       0
```
